# Dupilumab for Treatment of Prurigo Nodularis: Real-Life Effectiveness for up to 84 Weeks

**DOI:** 10.3390/jcm13030878

**Published:** 2024-02-02

**Authors:** Claudia Paganini, Marina Talamonti, Virginia Maffei, Cosimo Di Raimondo, Luca Bianchi, Marco Galluzzo

**Affiliations:** 1 Department of Systems Medicine, University of Rome “Tor Vergata”, 00133 Rome, Italy; cld.paganini@gmail.com (C.P.); virginiamaffei1@gmail.com (V.M.); luca.bianchi@uniroma2.it (L.B.); 2 Dermatology Unit, Fondazione Policlinico Tor Vergata, 00133 Rome, Italy; talamonti.marina@gmail.com (M.T.); cosimodiraimondo@gmail.com (C.D.R.)

**Keywords:** prurigo nodularis, dupilumab, atopic dermatitis

## Abstract

**(1) Background:** Prurigo nodularis (PN) is a persistent and inflammatory dermatological condition characterized by chronic itching and the formation of hardened nodules, significantly impacting the affected individuals’ quality of life and psychological well-being. The management of PN poses challenges due to the limited efficacy and undesirable side effects associated with current interventions. **(2) Methods:** This article examines sixteen patients affected by PN treated with dupilumab, a fully human monoclonal antibody targeting interleukin IL-4 and IL-13 signaling. This involves a retrospective descriptive statistical analysis. **(3) Results and (4) Conclusions:** In all patients, dupilumab proves to be an effective drug in achieving disease clearance, as indicated by all the parameters considered as assessed by both physicians and patients at each evaluation point (Week 6, Week 16, Week 32, Week 52, Week 68, and Week 84), in comparison to the initial baseline.

## 1. Introduction

Prurigo nodularis (PN) is a chronic inflammatory dermatological condition marked by persistent itching and the formation of indurated nodules. These manifestations have a substantial impact on the quality of life and psychological well-being of those affected. Managing prurigo nodularis presents difficulties, as many presently employed topical and systemic interventions show restricted efficacy and are associated with various undesirable side effects [1]. Dupilumab, a fully human monoclonal antibody that inhibits the common receptor component responsible for interleukin IL-4 and IL-13 signaling, has demonstrated success in addressing various dermatologic conditions. In this article, we explore the application of dupilumab in treating PN among adult patients. We present sixteen instances of dupilumab use in the treatment of PN. All individuals involved in this study had been dealing with prolonged and severe manifestations of the condition, having previously attempted various treatment approaches without significant success.

### Prurigo and Dupilumab

PN has been recently identified as a distinct disease entity, delineating it as a subtype separate from chronic prurigo (CPG). PN manifests with numerous nodules and papules that are symmetrically distributed and can reach up to hundreds in number. These skin formations are highly itchy (pruritus for at least 6 weeks), marked by hyperkeratosis, and may exhibit erosive or crusted features. It represents a prolonged response to the persistent scratching behavior of individuals suffering from chronic pruritus characterized by neuroimmune mechanisms [2,3]. The pathogenesis of PN remains incompletely understood. Prior research has shed light on the crucial role played by the interaction between nerve fibers in the skin and immune cells. Notably, there is evidence of diminished density of nerve fibers within the epidermis, coupled with elevated concentrations of neuropeptides like substance P, calcitonin gene-related peptide, and nerve growth factor in the dermal layers. Additionally, an abundance of eosinophils and mast cells is observed in this context [4]. The use of dupilumab has transformed the approach to managing PN, as evidenced by recent clinical trials showcasing its effectiveness in alleviating both itching and the development of nodules, while also enhancing overall quality of life [5]. The administration of dupilumab in the LIBERTY-PN PRIME and PRIME2 trials resulted in noteworthy enhancements across various facets of PN among patients with insufficiently managed conditions through topical therapies. The safety profile observed was in line with the previously established safety record of dupilumab. These favorable outcomes substantiate the implication of type 2 cytokines in steering the pathogenesis of PN and endorse the targeting of the IL-4/IL-13 axis as an innovative therapeutic approach for individuals with PN [5]. Thanks to the results of these trials, dupilumab is the first drug approved in the United States for the treatment of prurigo nodularis and received approval from the European Medicines Agency (EMA) for the same purpose in November 2022 [6].

## 2. Materials and Methods

### 2.1. Study Design

The overall aim of the study was to evaluate the clinical efficacy of dupilumab in patients with PN who were eligible for treatment with a biologic. 

Adult patients (≥18 years old) who were receiving monotherapy treatment at the Dermatology Unit of the Polyclinic Tor Vergata Foundation, Rome, Italy, were included. Data were collected from October 2019 to November 2023. Individuals undergoing concurrent treatment with systemic or topical therapies such as topical corticosteroids were not considered for inclusion in the study. 

The selection of participants for this retrospective, cross-sectional “snapshot” investigation relied on the examination of medical records, a process carried out by the clinicians responsible for this manuscript. Administration of dupilumab to patients with PN followed the Summary of Product Characteristics (briefly, an induction with a subcutaneous administration of 600 mg at Week 0 and a 300 mg dose maintenance administration every 2 weeks), including patients who did not respond to or showed contraindications or side effects to at least one conventional systemic therapy according to Italian law. PN was diagnosed following clinical criteria. At enrollment, clinical and demographic data, including age, sex, BMI, personal history of AD and/or other atopic manifestations, age at the onset of AD, duration of the disease, comorbidities, and information about both past and present therapies, were recorded. 

The evaluation of disease severity involved the use of several metrics: (a) the Investigator’s Global Assessment- Chronic Nodular Prurigo (IGA-CNPG) [7] score, which ranges from 0 to 4; (b) the itch Numeric Rating Scale (itch-NRS), with values from 0 to 10; (c) the sleeplessness Numeric Rating Scale (sleep-NRS), with scores ranging from 0 to 10; and (d) the Dermatology Life Quality Index (DLQI), which varies from 0 to 30. 

Prior to joining the study, every patient provided written consent expressing their willingness to participate. The study adhered to the ethical standards outlined in the 1975 Declaration of Helsinki. It is noteworthy that, in compliance with Italian regulations, formal approval from an ethical committee is not mandated for studies of this nature. [8] 

The efficacy of dupilumab treatment was evaluated by measuring IGA-CNPG, itch NRS, sleep NRS, and DLQI at Week 0, Week 6, and every 16 weeks thereafter. 

Different endpoints of response were considered: absolute IGA-CNPG score ≤ 1 achievement, itch-NRS score improvement ≥ 4 points, sleep-NRS score improvement ≥ 4 points, and DLQI improvement ≥ 4 points. Since the patients in this study commenced treatment at various points in time, the presented data should be viewed as a snapshot, providing a cross-sectional representation of our experience until November 2023.

### 2.2. Statistical Analysis

The data are expressed in the form of mean ± standard deviation (SD) for continuous variables, while categorical variables are presented as numbers and percentages. 

When data for an intermediate visit were missing, imputation was carried out using the last observation carried forward (LOCF) method. Statistical significance was established at *p* < 0.05. The analyses were conducted using SPSS (IBM SPSS Statistics for Windows, Version 27.0. Armonk, NY, USA: IBM Corp).

## 3. Results

In all, 16 patients with moderate-to-severe AD in the phenotype of PN were included in this study. Data were analyzed on 16 patients who were treated with at least four doses of dupilumab for >6 weeks. 

The mean age of the study population was 69 (SD 18.9) years; ten patients were female. Among the participants, eight patients had a history of cardiovascular disease, and two of these also presented with diabetes. Furthermore, two individuals had a history of chronic obstructive pulmonary disease (COPD), while one had idiopathic pulmonary fibrosis. Additionally, there was one patient with a history of platelet disorders, and two others with autoimmune disease. The mean disease duration was 21.6 (SD 16.8) years, and 2 patients had early-onset AD, while 14 had late-onset AD. The atopic comorbidities among the participants were as follows: three individuals reported a history of allergic rhinitis, two individuals had a documented history of asthma, and an additional three individuals exhibited symptoms consistent with allergic conjunctivitis. Patients were previously treated with the following: 16 instances of topical corticosteroids, 2 applications of topical calcineurin inhibitors, 8 administrations of antihistamines, 5 courses of systemic corticosteroids, and 3 instances of systemic drugs, which comprised either cyclosporine (CyA) or methotrexate (MTx). Additionally, one patient received phototherapy. 

Baseline demographic and clinical data are summarized in Table 1. 

**Figure 1:**
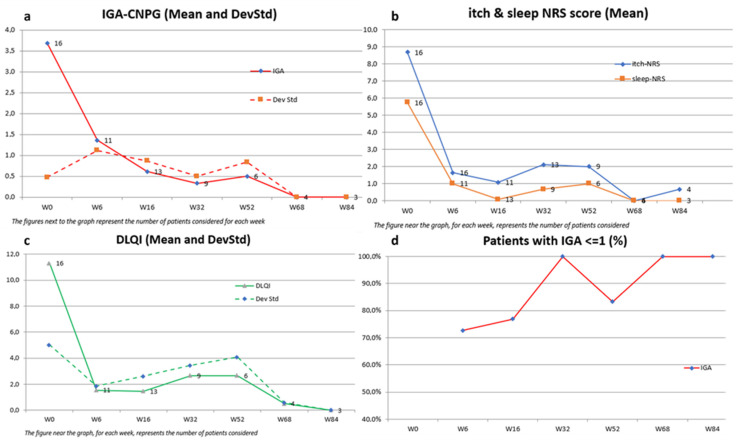
Effect of dupilumab on IGA-CNPG score, itch and sleep NRS, and DLQI over 84 weeks in patients with moderate-to-severe PN. Missing values for intermediate visits were imputed with the last observation carried forward (LOCF) method. (**a**) Mean IGA-CNPG scores in the overall population. (**b**) Mean itch and sleep NRS scores in the overall population. (**c**) Mean DLQI scores in the overall population. (**d**) Percentage of patients achieving an IGA score ≤ 1 over 84 weeks.

Dupilumab proved to be successful in achieving a substantial decrease in various outcome measures, as assessed by both physicians and patients at each evaluation point (Week 6, Week 16, Week 32, Week 52, Week 68, and Week 84), in comparison to the initial baseline (Figure 1). 

The mean IGA-CNPG score showed a significant reduction from 3.7 (SD 0.5) at baseline to 0.6 (SD 0.9) at Week 16, maintaining this clearance at Week 84 with a mean score of 0.0 (SD 0.0). This indicates a marked improvement in disease severity.

Similarly, the mean itch-NRS score, representing one of the most disabling symptoms, decreased from 8.7 (SD 1.7) to 0.7 (SD 1.1) over the 84-week period. This highlights a significant alleviation of itch symptoms, evident as early as Week 6, with a mean score of 1.6 (SD 2.2).

Sleep quality, as measured by the mean sleep-NRS score, improved from 5.8 (SD 2.5) to 0.0 (SD 0.0) by Week 84. Additionally, the mean DLQI score demonstrated a notable decline from 11.3 (SD 5) to 0.0 (SD 0.0) at Week 84, reflecting a substantial enhancement in dermatology-related quality of life.

Furthermore, a high percentage of patients achieved clinically meaningful improvements, with 100% reaching an IGA-CNPG score of 0/1 at Week 32 and Week 84, and significant proportions achieving ≥4-point amelioration in DLQI (67–100%), itch-NRS (78–100%), and sleep-NRS (50–100%) across various time points. 

Notably, all patients achieved a 4-point amelioration in the itch-NRS score by Week 16, while, during the same week, 85% and 92% reached a ≥4-point amelioration in sleep-NRS and DLQI scores, respectively. 

These findings underscore the efficacy of the intervention in ameliorating symptoms and enhancing the overall well-being of the study population (Table 2).

With regard to the safety profile of dupilumab, the most common side effects reported in the literature are reactions at the injection site and episodes of conjunctivitis [9].

In our study, only one patient developed an episode of conjunctivitis, which was treated solely with the use of eye drops.

## 4. Case Studies of Dupilumab Use in the Treatment of Prurigo Nodularis

### 4.1. Case 1

A 95-year-old man, with an history of platelet disorders, cardiovascular disease, and COPD, affected by PN from the age of 90, presented in April 2023 with severe PN, previously treated incorrectly for scabies and subsequently treated with topical corticosteroids and antihistamines with little benefit. Evaluation of IGA-CNPG was 4, and itch NRS score, sleep NRS score, and DLQI were 10, 10, and 15, respectively. 

**Figure 2:**
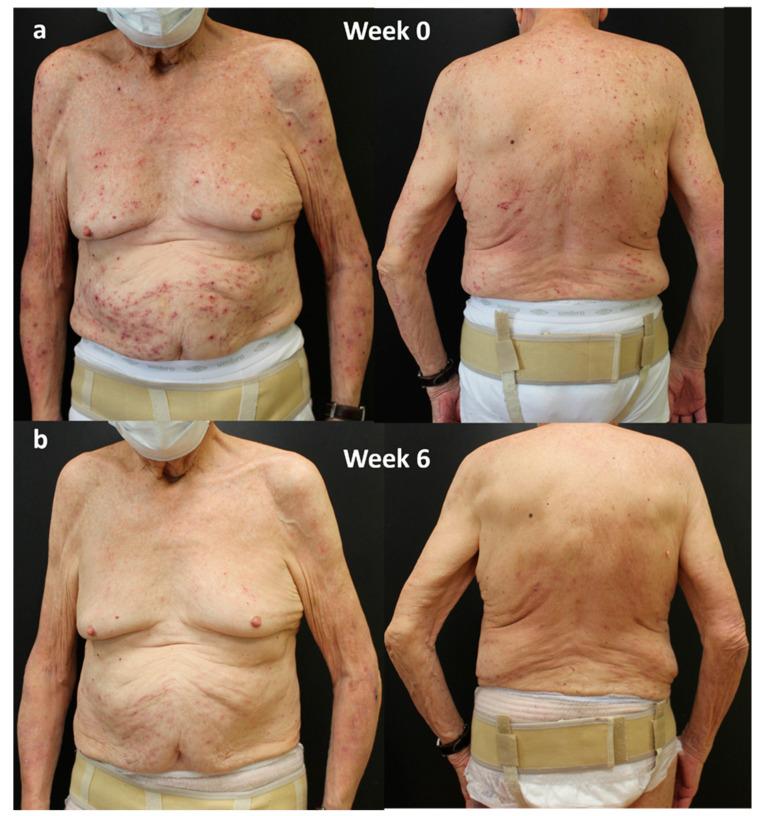
Response of a bio-naïve patient to dupilumab treatment over time: (**a**) patient at Week 0; (**b**) after 6 weeks of treatment. Consent to publish was obtained from the individual featured.

In April 2023, dupilumab was started. An excellent response was obtained in the first 6 weeks of treatment, highlighted by a clearing of disease parameters such as IGA-CNPG and itch and sleep NRS score and by a significant improvement in the quality of life (DLQI 0; Figure 2).

### 4.2. Case 2

A 69-year-old woman, with an history of diabetes, Hashimoto’s thyroiditis, and hypertension, affected by PN from the age of 25, presented in October 2020 with severe PN, previously treated with topical corticosteroids, antihistamines, and phototherapy (NB-UVB) with little benefit. Evaluation of IGA-CNPG was 4, and itch NRS score, sleep NRS score, and DLQI were 10, 8, and 15, respectively. 

**Figure 3:**
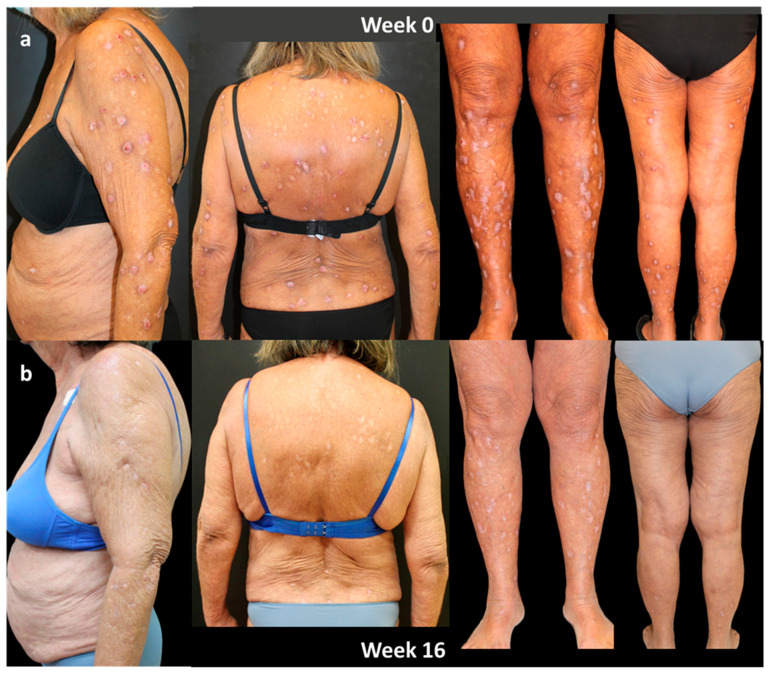
Response of a bio-naïve patient to dupilumab treatment over time: (**a**) patient at Week 0; (**b**) after 16 weeks of treatment. Consent to publish was obtained from the individual featured.

In October 2020, dupilumab was started. An excellent response was obtained in the first 16 weeks of treatment, highlighted by a clearing of disease parameters such as IGA-CNPG and NRS itch and sleep score and by a significant improvement in the quality of life. The patient is currently on 3 years of continuous treatment with dupilumab, and she is still in complete remission (both IGA-CNPG, itch and sleep NRS, and DLQI equal to 0; Figure 3).

### 4.3. Case 3

A 26-year-old man, with an history of allergic rhinitis, asthma, and conjunctivitis, affected by PN from the age of 22, presented in February 2019 with severe PN, previously treated with topical and systemic corticosteroids, with little benefit. Evaluation of IGA-CNPG was 4, and itch NRS score, sleep NRS score, and DLQI were 10, 10, and 20, respectively. 

**Figure 4:**
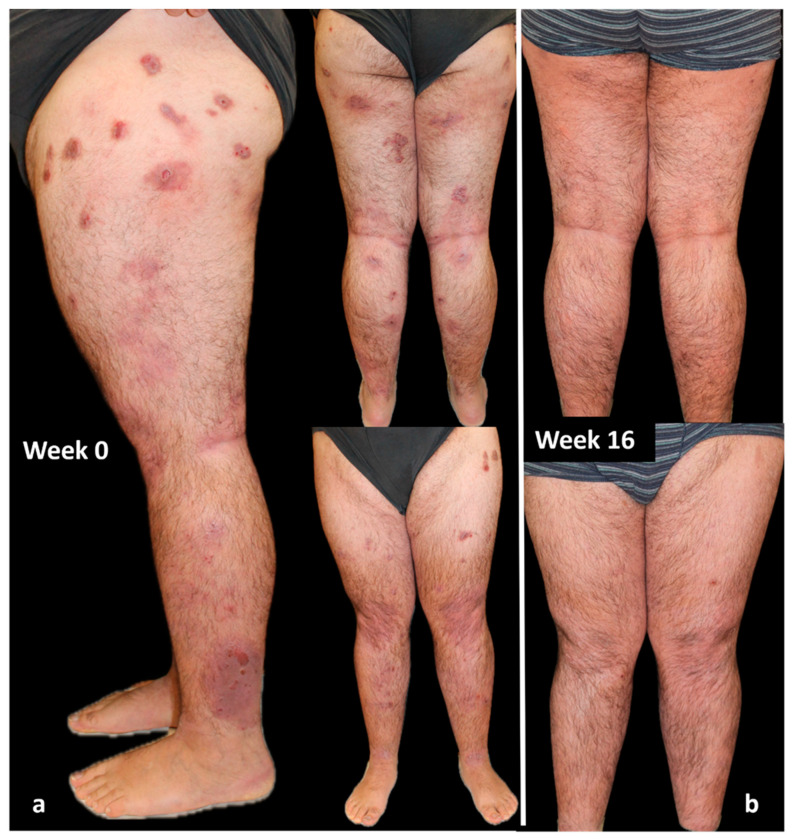
Response of a bio-naïve patient to dupilumab treatment over time: (**a**) patient at Week 0; (**b**) after 16 weeks of treatment. Consent to publish was obtained from the individual featured.

In March 2022, dupilumab was started. An excellent response was obtained in the first 16 weeks of treatment, highlighted by an almost clearing of disease parameters such as IGA-CNPG and NRS itch and sleep score and by a significant improvement in the quality of life. After 3 months of treatment, the patient developed an episode of conjunctivitis as a side effect of dupilumab. This condition was treated solely with the use of eye drops and resolved within 2 weeks. The patient is currently on 4 years of continuous treatment with dupilumab, and he is still in complete remission (both IGA-CNPG, itch and sleep NRS, and DLQI equal to 0; Figure 4).

## 5. Discussion

PN represents the prevailing subtype within the category of CPG [10]. It poses considerable challenges for both patients and physicians. Unlike many other medical conditions, there is a notable absence of large randomized controlled trials and treatment regimens approved by regulatory agencies specifically tailored for PN. Consequently, in the past, the therapeutic approach has often relied on off-label use of medications, indicating that treatments are prescribed for purposes other than those officially approved. This off-label nature of therapy in PN adds complexity to its management, requiring healthcare professionals to make informed decisions based on clinical experience and available evidence, while simultaneously highlighting the need for further research to establish evidence-based guidelines for the effective and safe management of this condition. 

The primary objectives of PN therapies were to disrupt the itch-scratch cycle, promote the healing of pruritic skin lesions, and alleviate pruritus. The approach involved addressing underlying conditions like dermatoses, cholestasis, diabetes, or nerve compressions. It was recommended to adopt a patient-individual stepwise approach, taking into account factors such as pruritus intensity, age, comorbidities, medication, accompanying strains (e.g., anxiety, depression), prior therapies, and potential side effects. Due to the complexity of cases, interdisciplinary cooperation was advised. 

**Figure 5:**
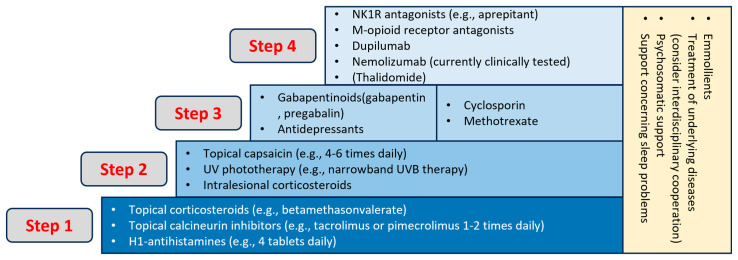
Treatment ladder of CNPG [15].

PN patients often necessitated a multimodal, long-term regimen comprising systemic and topical therapies, along with patient education and guidance. The initial international guideline recommended a laddered approach, incorporating topical treatments like corticosteroids and calcineurin inhibitors. Phototherapy and systemic treatments, including antihistamines, gabapentinoids, opioid receptor antagonists, antidepressants, immunosuppressants, small molecules, and biologics, were under consideration [11] (Figure 5).

Now, with the approval of dupilumab as a biologic for prurigo nodularis, the scenario has changed. The drug appears to be effective both in patients with a clinical history of atopic dermatitis and in patients who developed prurigo nodularis in adulthood without a history of atopy.

In our study, dupilumab proves to be effective in treating patients with nodular prurigo, reducing all disease parameters, particularly leading to the resolution of some nodules ulcerated by systematic scratching, thanks to its rapid action on itching (at Week 6, 100% of patients achieved an improvement in itch NRS ≥ 4 points). It is indeed the symptom of itching that patients perceive as most disabling, as evidenced by the fact that with the improvement of itching, there is a simultaneous improvement in the quality of life of patients, as reflected in the DLQI, which is already an average of 1.5 in all considered patients at Week 6. 

Another comorbidity directly correlated with nodular prurigo is sleep disturbance due to pruritic symptoms. In this case as well, the sleep NRS value showed an improvement of at least 4 points already by Week 6 in 82% of patients. 

Improvements in symptoms correlated with a decrease in erythema and infiltration of chronic nodular prurigo lesions. This was evidenced by an increasing number of patients exhibiting a lowered IGA-CNPG score throughout the entire observation period. Additionally, a score of IGA-CNPG 0/1 was achieved by all patients at Week 32 and Week 84. Overall, treatment-emergent adverse events consisted of one case of mild conjunctivitis treated with eye drops.

As in our study, the experience of Zhang et al., analyzing 8 patients with nodular prurigo for more than 6 months, also indicates that at the 16th week of dupilumab treatment, two patients (25.0%) achieved a full response, showing a reduction in IGA by 3 grades. Half of the population (50.0%) experienced a partial response, indicating a decrease in IGA by 2 grades [12].

In their study, Calugareanu et al. observed that 50% of patients with PN achieved a complete response, 41.7% experienced a partial response, and 1 out of 16 patients showed no response after six months of dupilumab treatment [13].

In addition, Chiricozzi et al. analyzed a cohort of 27 CNPG patients, and dupilumab was proven effective in reducing itch and improving CPNG skin lesions. At Week 4, there was a reduction in IGA score in 51.8% of patients. A considerable decrease in both itch and sleep NRS values was observed, with values dropping from 8.9 to 6.3 for itch and from 8.2 to 5.5 for sleep, respectively (*p* values < 0.001). This significant improvement was noted in the majority of patients and was concurrently linked with a reduction in the DLQI score, declining from 21.0 to 11.8 (*p* < 0.001) [14].

The evidence presented above strongly advocates for the efficacy of dupilumab in the treatment of PN, whether it is associated with atopic comorbidities or not. In our study, there was no discernible difference in the treatment response between patients with atopic march and PN and those with no atopic comorbidities and PN. This subgroup, constituting half of our study population (8 out of 16), displayed comparable responses to dupilumab treatment.

In this cohort of adult patients with PN, the administration of dupilumab has demonstrated safety, with no reported severe adverse events (SAE). Previous instances of dupilumab treatment in PN cases have also supported its safety, as no SAE were identified. The most common adverse events include reactions at the injection site and episodes of conjunctivitis [9]. Less common adverse events such as paradoxical head and neck erythema, psoriasis, alopecia, and arthritis were mentioned, but none were detected in the cited studies. The findings from this study suggest that dupilumab is an effective and well-tolerated treatment option for adults dealing with persistent and challenging PN.

Furthermore, its approval for the treatment of pediatric atopic dermatitis once again underscores its safety profile. This suggests its potential use in the elderly with multiple comorbidities, as revealed in our study. The versatility and safety of dupilumab make it a promising option across different age groups, offering a valuable therapeutic solution. In the wake of dupilumab, other therapies approved for atopic dermatitis could also prove effective in treating prurigo. For instance, Janus kinase inhibitors are currently under exploration, and ongoing clinical trials are assessing novel molecules in both the United States and Europe [10].

Continued exploration of the pathogenesis of prurigo nodularis is imperative, with a specific emphasis on investigating the bidirectional interplay between peripheral and central neuroimmunological mechanisms. A comprehensive understanding of these intricate processes is indispensable for the advancement of innovative and efficacious multimodal treatment strategies. This research not only holds importance in elucidating the complexities associated with prurigo nodularis but also has the potential to significantly elevate the standard of future patient care by offering more precise and comprehensive therapeutic interventions.

## 6. Conclusions

The PN appears to be a phenotype of adult AD even in those patients with no history of atopy. It is a chronic skin disease that significantly impairs patients, as evidenced by the reported DLQI values in case studies. Itching, the major symptom of PN, is perceived as particularly disabling in the general population, as indicated in the relevant literature. It has the potential to compromise the well-being of individuals across all age groups, including young, adult, and especially elderly individuals. Despite the ability of PN to affect people of all ages, the elderly are particularly vulnerable to its impact. Unfortunately, these elderly patients often present a complex clinical history characterized by multiple comorbidities, necessitating treatment with various medications. The challenge in diagnosis becomes evident as healthcare professionals must diligently exclude all potential internal causes of itching [15]. Moreover, the prescribed therapy should not interact adversely with other medications. Indeed, 75% of patients in our study were over 65 years old, and 100% of this subgroup had a clinical history characterized by multiple comorbidities under treatment.

In this context, dupilumab, a biological drug with an excellent safety profile, proves advantageous. It not only reduces the risk of interactions but also minimizes the occurrence of serious side effects in patients with multiple comorbidities.

Indeed, even from our experience, it emerges that in our sample of patients, 87.5% were over 65 years old, and they responded excellently to dupilumab, with IGA ≤ 0/1 achieved by all patients starting from the 32nd week and a significant alleviation of itch symptoms, evident as early as Week 6, with a mean score of 1.6 (SD 2.2).

## Figures and Tables

**Table 1:** Demographic.

Total Population	16
Age (years; mean ± SD)	69 ± 18.9
M/F	6/10
BMI (mean ± SD)	23.8 ± 3.6
Comorbidities	
Cardiovascular disease	8
Diabetes	2
COPD	2
Idiopathic pulmonary fibrosis	1
Platelet disorders	1
Autoimmune disorders	2
Course of disease	
Duration of disease (mean ± SD)	21.6 ± 16.8
Early-onset AD (<18 years old)	2
Late-onset AD (>18 years old)	14
Allergic comorbidities	
Allergic rhinitis	3
Asthma	2
Allergic conjunctivitis	3
Previous treatments	
Topical corticosteroids	16
Topical calcineurin inhibitors	2
Antihistamines	8
Systemic corticosteroids	5
Systemic drugs (CyA o MTx)	3
Phototherapy	1

**Table 2:** Results.

	Overall Population						
	Baseline	Week 6	Week 16	Week 32	Week 52	Week 68	Week 84
IGA-CNPG score (mean ± SD)	3.7 ± 0.5	1.4 ± 1.1	0.6 ± 0.9	0.3 ± 0.5	0.5 ± 0.8	0.0 ± 0.0	0.0 ± 0.0
itch-NRS score (mean ± SD)	8.7 ± 1.7	1.6 ± 2.6	1.1 ± 1.6	2.1 ± 2.9	2 ± 3.1	0.0 ± 0.0	0.7 ± 1.1
sleep-NRS (mean ± SD)	5.8 ± 2.5	1.0 ± 2.2	0.1 ± 0.2	0.7 ± 1.4	1.0 ± 1.5	0.0 ± 0.0	0.0 ± 0.0
DLQI score (mean ± SD)	11.3 ± 5	1.5 ± 1.9	1.5 ± 2.6	2.7 ± 3.4	2.7 ± 4.1	0.5 ± 0.6	0.0 ± 0.0
N.(%) patients reaching IGA-CNPG 0/1		72.7%	76.9%	100%	83%	100%	100%
N.(%) patients reaching ≥4-point amelioration DLQI		82%	92%	100%	83%	75%	67%
N.(%) patients reaching ≥4-point amelioration itch—NRS		91%	100%	78%	83%	100%	100%
N.(%) patients reaching ≥4-point amelioration sleep—NRS		82%	85%	67%	50%	100%	100%

## Data Availability

The original contributions presented in the study are included in the article, further inquiries can be directed to the corresponding author.
